# Complete Chloroplast Genome Characterization, and Phylogenetic Analyses of the Rare and Endangered Plant *Platycrater arguta*

**DOI:** 10.3390/biology14121726

**Published:** 2025-12-01

**Authors:** Xiaohua Ma, Youju Ye, Ren’an Lin, Qingdi Hu, Xule Zhang, Yaping Hu, Lei Feng, Renjuan Qian, Jian Zheng

**Affiliations:** 1 Wenzhou Key Laboratory of Resource Plant Innovation and Utilization, Engineering Research Center for Southeast Coastal Characteristic Plants of National Forestry and Grassland Administration, Zhejiang Institute of Subtropical Crops, Wenzhou 325005, China; 2 Yueqing Forestry Seed Science and Technology Center, Yueqing 325699, China

**Keywords:** *Platycrater arguta*, chloroplast genome, phylogenetic analysis, evolution relationship

## Abstract

As an important rare and endangered plant, *Platycrater arguta* urgently requires protective measures. In this study, we assembled and annotated a more complete chloroplast genome of *P. arguta*, which has a size of 157,812 bp. The chloroplast genome comprises a large single-copy (LSC) region of 86,825 bp, a small single-copy (SSC) region of 18,735 bp, and a pair of inverted repeats (IRa and IRb) of 26,126 bp each. SSR detection revealed that the chloroplast genome contains 199 simple repeat loci, with mononucleotide repeats of A and T being the most frequent, accounting for 59.8%. Comparative analysis of the chloroplast genome structure showed that the IR region boundaries are relatively conserved. The results of the phylogenetic tree indicate that *P. arguta* shares high homology with Hydrangea davidii. This work provides a scientific basis for future research on the genetics, biology, and conservation of *P. arguta*.

## Simple Summary

We assembled and annotated a more complete chloroplast genome of *P. arguta*, which includes an 86,825 bp large single-copy (LSC) region, an 18,735 bp small single-copy (SSC) region, and two 26,126 bp inverted repeats (IRa/IRb). SSR detection identified 199 simple repeat loci, with A/T mononucleotide repeats being most frequent (59.8%). Phylogenetic tree results revealed high homology between *P. arguta* and *Hydrangea davidii*. This work provides a scientific basis for future studies on *P. arguta’s* genetics, biology, and conservation.

## 1. Introduction

Chloroplasts are plant-specific organelles responsible for photosynthesis, through which plants convert light energy into chemical energy and store it in cells [1]. In addition, chloroplasts also play a vital role in plant growth and development, as they are crucial for plant responses to light, heat, drought, salt, and other abiotic stresses [2]. The chloroplast genome encodes several important proteins that participate in metabolic processes, and the genes within this genome exhibit a relatively high degree of conservation across plant species [3,4]. Furthermore, the chloroplast genome exhibits a low recombination rate, which results in less genetic variation within itself (compared to the nuclear genome). This characteristic makes it widely applicable in phylogenetic analysis of plants at different taxonomic levels [5,6].

The chloroplast genomes are highly conserved in their overall organization in most lineages, with sizes ranging from <100 bp to >1000 bp, and typically have a four-part structure, including two inverted repeat (IR) regions, one large-single copy (L) region, and one small single-copy (SSC) region [7,8]. However, significant variations in the size, structural arrangement, and gene content of chloroplast genomes have been recorded from algae to angiosperms, reflecting evolutionary trajectories in these organisms [8]. For example, some algal plastid genomes contain large amounts of non-coding DNA, which occupies a part of the genome and may be involved in regulating gene expression, or in the replication and repair of the genome. Others are filled with autosplicing introns, as in *Chlamdomonas* [8]. The number of genes in the chloroplast genome is significantly fewer than that in the nuclear genome [7]. Due to variations in evolutionary patterns and geographic distribution, the chloroplast genomes of different plant groups may exhibit differences in gene presence/absence, repeat sequences, deletions, rearrangements, and other structural variations [9].

The rapid advancement of next-generation sequencing (NGS) technology has significantly accelerated research progress in chloroplast genetics and genomics, enhancing both the efficiency (speed) and depth of such studies [10]. Prior to the widespread adoption of NGS, *Nicotiana tabacum* (tobacco) was the first species for which a complete chloroplast genome was sequenced, with this milestone achieved in 1986 [11]. Subsequently, complete chloroplast genome sequences were generated for numerous other species, including *Marchantia polymorpha* [12], *Oryza sativa* [13], *Euglena gracilis* [14], and *Pinus thunbergii* [15]. To date, over 2000 complete chloroplast genome sequences have been publicly submitted to the National Center for Biotechnology Information (NCBI) database. The continuous expansion of chloroplast genome data in such public repositories will make valuable contributions to phylogenetic inference and related evolutionary studies [16,17]. For example, Hu et al. (2023) reported that the complete chloroplast genome of *Sphaeropteris lepifera*, a rare and endangered tree fern, has a length of 162,150 base pairs (bp), and they identified 161 simple sequence repeat (SSR) loci within this genome [10]. Phylogenetic analysis based on the complete chloroplast genome further indicated that *Sphaeropteris lepifera* shares a closer evolutionary relationship with *Alsophila podophylla* than with other related genera. Separately, Huo et al. (2019) compared the chloroplast genomes of nine *Allium* species, demonstrating the conservation of these genomes in terms of genome structure and gene organization; additionally, phylogenetic tree analysis clustered these nine *Allium* species into two distinct clades [18].

*Platycrater arguta*, a member of the family Hydrangeaceae and the monotypic genus Platycrater (i.e., a genus containing only one species), is a relic plant that existed in Japan prior to the archipelago’s separation from the Asian continent [19]. This deciduous shrub, endemic to East Asia, holds substantial value for investigating the phylogeography of continental-island disjunct distributions, a key biogeographic pattern in evolutionary ecology. Notably, in 1992, it was listed in the “Red Data Book of China” and designated as a national second-class endangered species under state protection, reflecting its critical conservation status [19].

*P. arguta* flowers in summer, producing white blooms with an elegant morphology; their petals exhibit dense venation resembling a spider’s web, hence its common name, “spiderweb calyx”. Owing to its unique floral architecture, high esthetic appeal, tolerance to pruning, and facile shaping, this species is widely utilized in flower beds and as indoor potted plants to enhance residential and horticultural environments, conferring substantial ornamental and economic value [19]. Furthermore, its leaves can serve as a tea substitute, exhibiting purported health-promoting effects, including physical vitality enhancement and mental refreshment, which signifies promising development prospects for its sustainable utilization. However, in recent decades, wild populations of *P. arguta* have declined drastically, and its distribution range has become progressively fragmented [19], a trend that threatens its long-term survival. To mitigate this adverse scenario, it is imperative to implement targeted protective measures for *P. arguta*, such as habitat restoration, ex situ conservation, and population monitoring, to safeguard its genetic diversity and ecological function.

Studies on chloroplast genomes are integral to the broader genomic research framework, and the chloroplast genome of *P. arguta* (hereafter *P. arguta*) provides a critical foundation for analyzing the species’ genetic information (particularly chloroplast-derived data) and supporting its germplasm conservation. However, current genomic resources for *P. arguta* remain limited, and previous chloroplast genome-related studies of this species are either incomplete or lack systematic comparative analysis, hindering in-depth investigations into its evolutionary biology and conservation needs. This study aims to (i) assemble and annotate the complete chloroplast genome of *P. arguta*, (ii) analyze its structural and evolutionary features, and (iii) infer phylogenetic relationships within Saxifragaceae. To achieve these objectives, we performed assembly, annotation, and comprehensive feature analysis of the *P. arguta* chloroplast genome, and inferred the phylogenetic relationships among 22 Saxifragaceae species. The results of this research are expected to serve as a robust reference for future studies on *P. arguta*, including species authentication, the development of ecological conservation strategies, and the elucidation of drivers underlying its endangered status.

## 2. Materials and Methods

### 2.1. Plant Material

The leaves of *P. arguta* were collected from Yandang Mountain, Wenzhou City, Zhejiang Province (27°50′ N, 120°27′ E). Total genomic DNA was extracted using a DNA Extraction Kit (Tiangen, Beijing, China) and stored at −80 °C until subsequent use [20]. Genomic DNA was then fragmented via mechanical disruption (ultrasound), followed by fragment purification, end repair, 3′-end adenylation, and ligation of sequencing adapters. Fragment size selection was performed using electrophoresis. The sequencing library was subsequently constructed through PCR amplification and subjected to paired-end (PE) sequencing on the Illumina NovaSeq 6000 platform (Illumina, Inc, San Diego, CA, USA).

### 2.2. The Sequence, Assembly and Annotation of Chloroplast Genome Sequence in P. arguta

Raw sequencing data were filtered using Fastp software (version 0.20.0, https://github.com/OpenGene/fastp) (accessed on 10 December 2023) [19]. High-quality clean data were obtained following these criteria: (1) removal of sequencing adapters and primer sequences from reads; (2) filtering out reads with an average quality score below Q20 or a length shorter than 50 bp; and (3) excluding reads containing more than 5 ambiguous “N” bases.

To acquire aligned sequences representing the chloroplast genome (cpDNA) of *P. arguta*, the very-sensitive-local algorithm of Bowtie2 (v2.2.4, https://bowtie-bio.sourceforge.io/bowtie2/index.shtml (accessed on 15 December 2023) was employed to map reads against a chloroplast genome database constructed by Nanjing Genepioneer Biotechnologies Co., Ltd., Nanjing, China [21].

Chloroplast genome assembly was performed using SPAdes v3.10.1 software (http://cab.spbu.ru/software/spades/ (accessed on 17 December 2023) [22], with kmer used 55, 87, and 121; and the assembly did not depend on the reference genome; following a seven-step protocol: SPAdes initially assembled cpDNA sequences to generate seed sequences; if contigs were produced, they were designated as pseudogenome sequences and processed directly in step 6; when contigs remained unassembled, SSPACE v2.0 https://bioinformaticshome.com/tools/wga/descriptions/SSPACE.html#gsc.tab=0 (accessed on 19 December 2023) was utilized to connect contigs into scaffolds; GapFiller v2.1.1 (https://sourceforge.net/projects/gapfiller/ (accessed on 21 December 2023) was applied to correct gaps within these scaffolds; if gaps persisted, specific primers were designed for PCR amplification and subsequent reassembly until complete pseudogenome sequences were obtained; sequences were aligned against the pseudogenome for genome correction; the corrected pseudogenome coordinates were rearranged according to canonical chloroplast genome structural features to generate circular chloroplast genome sequences [23].

To ensure accurate annotation of the *P. arguta* chloroplast genome, a two-pronged approach was implemented—integrating gene-specific tools with commonly used chloroplast genome annotation platforms: Coding sequences (CDS) were annotated using Prodigal v2.6.3 (https://www.github.com/hyattpd/Prodigal (accessed on 22 December 2023), while rRNAs and tRNAs were predicted using HMMER v3.1b2 (http://www.hmmer.org/ (accessed on 23 December 2023) and Aragorn v1.2.38 (https://bioconda.github.io/recipes/aragorn/README.html (accessed on 25 December 2023), respectively; annotations were further cross-referenced with results from the GeSeq platform to validate consistency. BLAST v2.6 (https://blast.ncbi.nlm.nih.gov/Blast.cgi (accessed on 26 December 2023) was used to align assembled sequences against published gene sequences of closely related species from the NCBI database (https://www.ncbi.nlm.nih.gov/) (accessed on 26 December 2023) [24]. The annotation datasets were manually cross-validated to resolve discrepancies, with erroneous and redundant annotations removed. Exon boundaries were precisely determined to finalize the annotation.

### 2.3. Chloroplast Genome Analysis

The codon usage bias of the *P. arguta* chloroplast genome was analyzed using the CodonW online tool integrated within the GALAXY platform (https://galaxy.pasteur.fr (accessed on 28 December 2023) [25]. To minimize deviations in codon usage bias results, protein-coding genes shorter than 300 nucleotides were excluded from the analysis.

Simple sequence repeats (SSRs) were identified across seven chloroplast genomes of Sapindaceae species using the MISA online tool (https://webblast.ipk-gatersleben.de/misa/ (accessed on 30 December 2023), with the following repeat unit threshold settings: 10 repeats for mononucleotides, 5 repeats for dinucleotides, 4 repeats for trinucleotides, and 3 repeats for tetra-, penta-, and hexanucleotides [26,27]. Repetitive sequences, encompassing forward, reverse, palindromic, and complementary repeats, were detected via the REPuter program vmatch 2.3.0, with parameters set to a minimum repeat region length of 10 bp and a minimum sequence identity of 90% [28].

The expansion and contraction of inverted repeat (IR) regions at junction sites were investigated across seven Sapindaceae chloroplast genomes, specifically those of *H. davidii* (MT861130.1), *Hydrangea petiolaris* (NC_034935.1), *Hydrangea moellendorffii* (NC_044805.1), *Kirengeshoma palmata* (NC_044808.1), *Hydrangea paniculata* (NC_044829.1), and *Deutzia compacta* (NC_044843.1). These IR junction dynamics were examined and visualized using the IRscope online tool (https://irscope.shinyapps.io/irapp/ (accessed on 30 December 2023) [29].

### 2.4. Phylogenetic Tree Was Conducted by the Chloroplast Genome Sequence

To clarify the phylogenetic position of *P. arguta* within Saxifragaceae and ensure representative taxon sampling, chloroplast genome sequences were retrieved from the NCBI database following three criteria: (1) 18 Saxifragaceae species covering 8 major genera (e.g., *Hydrangea*, *Kirengeshoma*, *Deutzia*) to represent the family’s taxonomic diversity; (2) 3 outgroup taxa (*Nasa triphylla*, *Caiphora lateritia*, *Mentzelia albicaulis*) from closely related families (Loasaceae) based on previous angiosperm phylogenetic frameworks [30], ensuring robust root placement; and (3) inclusion of *P. arguta* for direct phylogenetic positioning. In total, 22 taxa were analyzed. Complete chloroplast genome sequences of these taxa were first aligned using MAFFT v7.511 (https://mafft.cbrc.jp/alignment/server/ (accessed on 30 December 2023). Subsequent phylogenetic tree construction was performed in RAxML v8.2.10 (https://cme.h-its.org/exelixis/software.html (accessed on 30 December 2023) under the GTRGAMMA substitution model, with 1000 bootstrap replicates to assess branch support [31].

## 3. Results

### 3.1. Characteristics of the Chloroplast Genome in P. arguta

**Figure 1:**
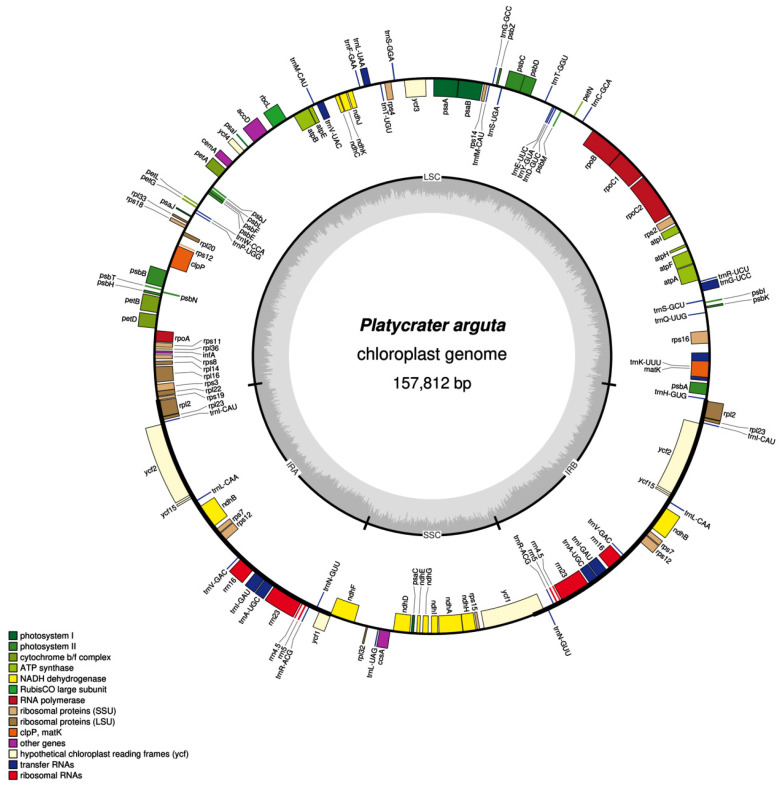
Diagram of chloroplast genome in *P. arguta*. The transcribed clockwise and counterclockwise was drowned outside and inside, respectively. Genes with different functions are represented by different colors. The GC content was represented by the darker gray, and the AT content was represented by the lighter gray shows.

The chloroplast genome of *P. arguta* exhibits the typical quadripartite structure characteristic of most angiosperms. The size of the chloroplast genome in *P. arguta* is 157,812 bp. It consists of a large single-copy (LSC) region of 86,825 bp, a small single-copy (SSC) region of 18,735 bp, and a pair of inverted repeats (IRs) each measuring 26,126 bp. The genome encodes a total of 133 genes, including 88 protein-coding genes (PCGs), 37 transfer RNA (tRNA) genes, and 8 ribosomal RNA (rRNA) genes. Intron analysis reveals that three genes (*ycf3*, *clpP*, and *rps12*) contain two introns, while fifteen unique genes (*trnA-UGC*, *trnG-UCC*, *trnI-GAU*, *trnK-UUU*, *trnL-UAA*, *trnV-UAC*, *ndhA*, *ndhB*, *petB*, *petD*, *atpF*, *rpl16*, *rpl2*, *rps16*, and *rpoC1*) each contain a single intron (Figure 1, Table 1).

### 3.2. Sequence Analysis

**Figure 2:**
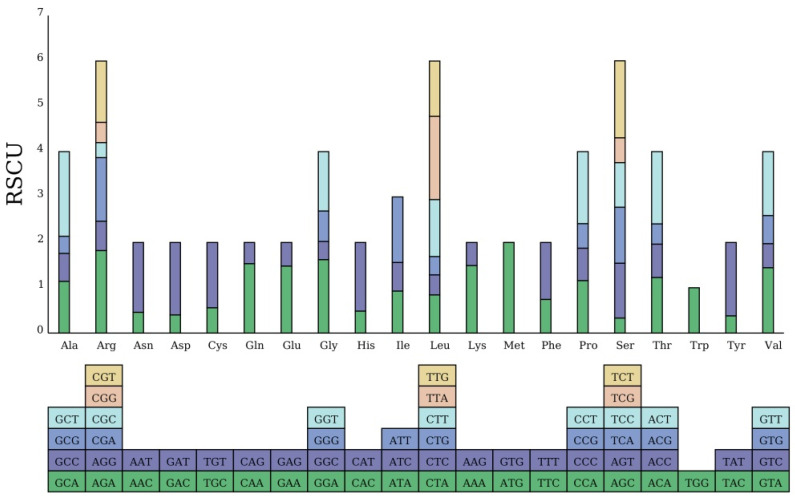
Chloroplast genome sequence codon usage bias. The sum of the RSCU value of all the codons is represented by the height of the upper column, and all codons encoding each amino acid are represented by boxes below. In addition, the codons are represented by different colors.

A total of 26,920 codons were identified across all protein-coding sequences (PCSs). Leucine was the most frequently occurring amino acid, accounting for 2840 instances, whereas termination codons (Ter) were the least common, with only 88 occurrences. To further quantify codon usage bias, we calculated two key metrics: the GC content of the third codon position (GC3) was 28.6%, indicating a strong A/T preference at synonymous sites; and the Effective Number of Codons (ENC) was 47.2, suggesting moderate codon usage bias (ENC ranges from 20, extreme bias, to 61, no bias). Tryptophan, encoded exclusively by the codon TGG, exhibited no codon usage bias, as indicated by a relative synonymous codon usage (RSCU) value of 1. Notably, 65 codons had an RSCU value > 1, demonstrating significant codon usage bias (Figure 2). Specifically, among the codons with usage bias in *P. arguta*, 63 were A/T-ending. The only exceptions were the leucine-encoding codon UUG and the methionine-encoding codon AUG, both of which were G-ending; details of these codons are presented in Figure 2.

**Figure 3:**
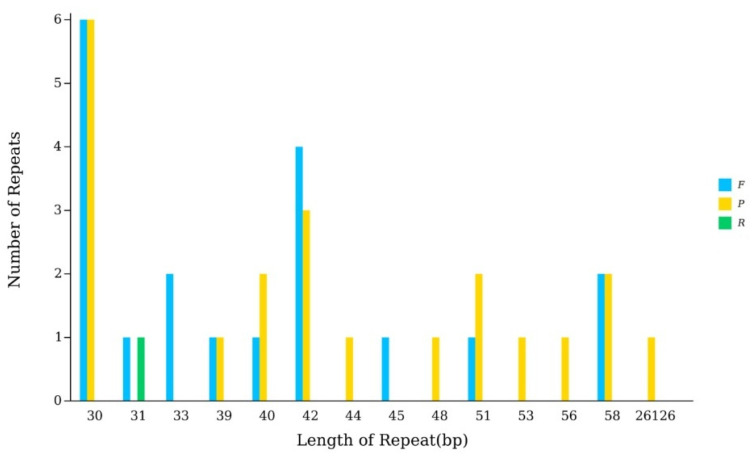
The chloroplast genome repeated sequences in *P. arguta.* In detail, the X-ray are the length of the repeated sequence, and the Y-ray are the number of repeated sequences. F represents Forward, P stands for Ralindromic, R represents Reverse, and C represents Complement.

In the chloroplast genome of *P. arguta*, a total of 40 larger repeats (>10 bp) were identified, comprising 19 forward repeats, 1 reverse repeat, and 21 palindromic repeats (Figure 3, Table S1). Notably, the largest of these repeats was a 26,126 bp palindromic repeat, which corresponds to the inverted repeat (IR) region, a canonical structural feature of angiosperm chloroplast genomes.

**Figure 4:**
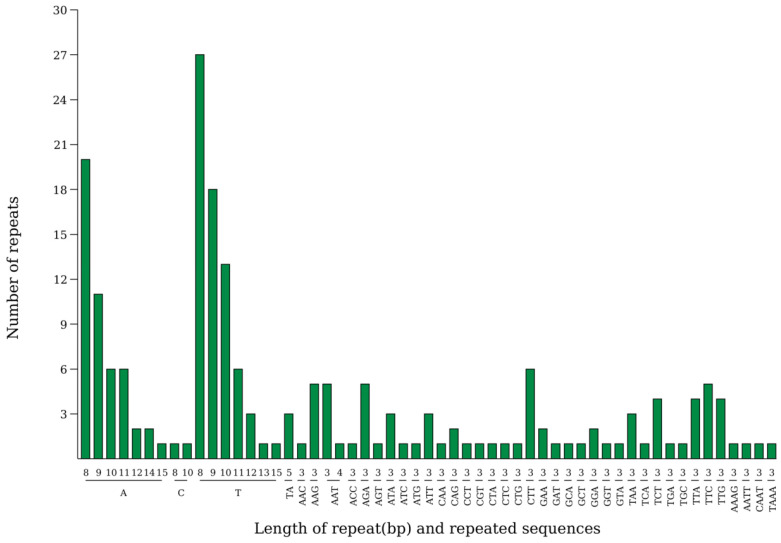
The SSRs distribution in the chloroplast genome of *P. arguta*. X-ray represents SSR repeated unit, and the Y-ray represents the number of repeated units.

This study analyzed the simple sequence repeats (SSRs) located in the large single-copy (LSC), inverted repeat (IR), and small single-copy (SSC) regions, as well as in intergenic spacer (IGS) and gene regions. The highest number of repeats was found in the LSC region (62.30%), followed by the IR regions (19.10%) and the SSC region (18.60%). A total of 199 SSRs were identified in the chloroplast genome of *P. arguta* in the present study. Among these, 119 were mononucleotides, 3 were dinucleotides, 73 were trinucleotides, and 4 were tetranucleotides (Figure 4).

**Figure 5:**
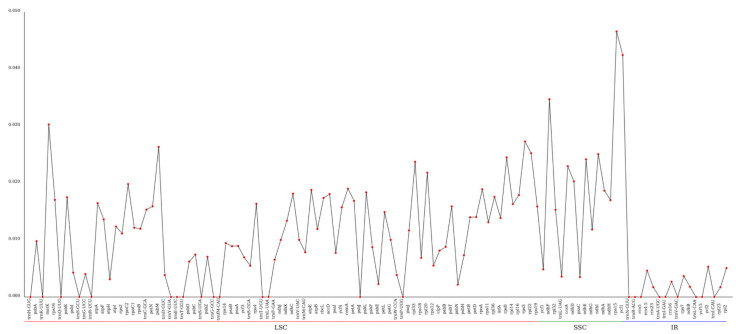
The nucleotide diversity (Pi) analysis of the chloroplast genome among 7 species. The X-ray represents the gene name, while Pi value is presented by the Y-ray.

The average nucleotide diversity (Pi) of the chloroplast genome was calculated to be 0.01058. Notably, there were 37 regions exhibiting significantly higher Pi values (>0.01537). Among these, the *rps*15 gene region demonstrated the highest variability, with a Pi value of 0.04656, located within the SSC region (Figure 5).

### 3.3. Expansion and Contraction of IR Regions

**Figure 6:**
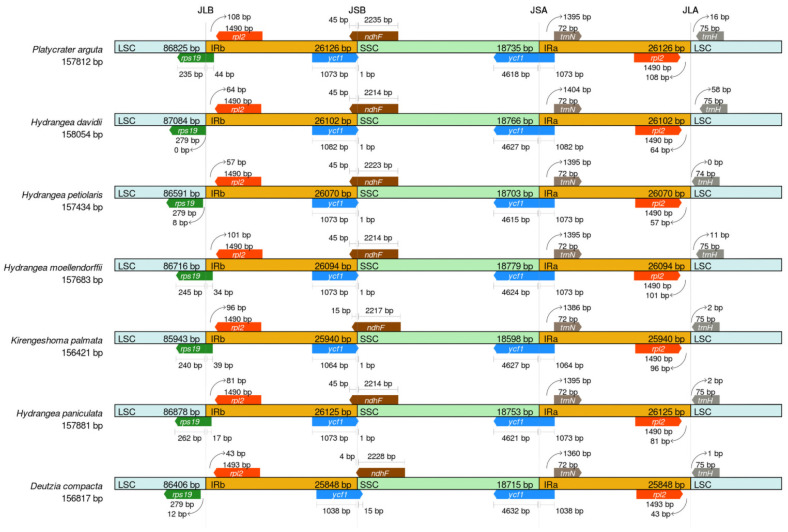
Comparative analysis of the LSC, SSC and IR regions in chloroplast genomes among seven Saxifragaceae.

To analyze the evolutionary conservation of the Saxifragaceae, the IR regions and junction sites of the LSC and SSC regions were compared based on the chloroplast genomes of seven members of the Saxifragaceae family, including *P. arguta* (Figure 6). The length of the IR regions varied, ranging from 25,848 bp in *D. compacta* to 26,126 bp in *P. arguta*. A striking feature of the *P. arguta* chloroplast genome is its IR configuration: unlike the canonical arrangement where certain genes are typically fully contained within IR regions, the protein-coding gene *ycf1* is largely excluded from the IRs, with only a very small portion present in these repeats. This deviation from the typical structure is noteworthy and may reflect unique evolutionary dynamics of the *P. arguta* chloroplast genome. In the present study, the *ycf1* gene was found in the SSC/IRa junction across all seven species, while the LSC/IRa junction was located downstream of *trnH-GUG*. Additionally, the coding region of *rpl22* created locations of 44, 0, 8, 34, 39, 17, and 12 bp at the LSC/IRb border, with the LSC/IRb junction distributed, respectively, across all seven chloroplast genomes.

### 3.4. Phylogenetic Analysis

**Figure 7:**
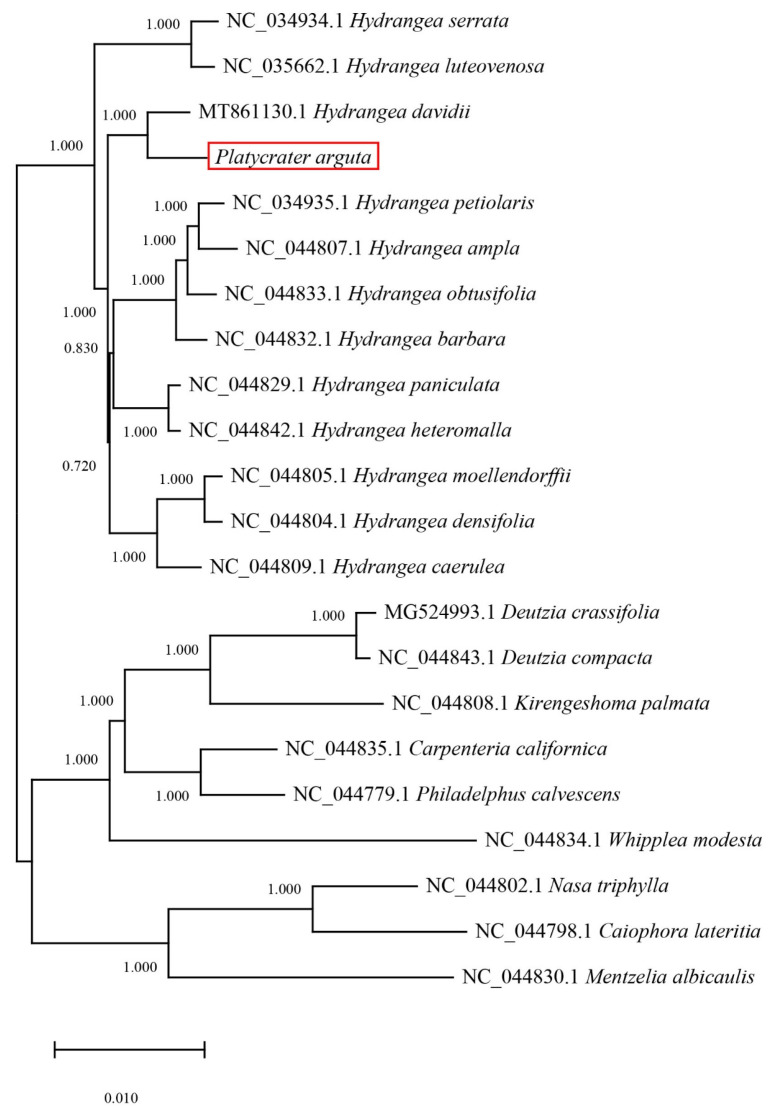
The result of phylogenetic tree in 21 species. The phylogenetic tree was constructed based on the chloroplast genome with the GTRGAMMA model in RAxML v8.2.10, and bootstrap replicated 1000 times.

Multiple sequence alignments of 21 species based on chloroplast genomes were conducted. Specifically, 18 species belong to the Saxifragaceae family, while 3 outgroup species include *Nasa triphylla*, *Caiphora lateritia*, and *Mentzelia albicaulis*. The results indicated that *P. arguta* exhibited the highest homology with *H. davidii*, followed by the other 17 Saxifragaceae species. Furthermore, the outgroup species demonstrated lower homology with *P. arguta* (Figure 7).

## 4. Discussion

Chloroplasts are plant-specific organelles that replicate independently and exhibit their own genetic inheritance. They play a crucial role in various functions within plant cells, such as photosynthesis, carbon fixation, and stress responses [32,33]. The chloroplast genome has a relatively small and highly conserved molecular structure, typically ranging from 107 to 218 kb in length, and encodes approximately 80 proteins [34]. Due to their relatively stable evolutionary rate and low nucleotide substitution rates, chloroplast genomes are widely utilized in plant research, providing valuable resources for species identification, genetic engineering, and phylogenetic analyses [34].

As the plant-specific organ, chloroplasts provide abundant genetic resources that are crucial for the protection of *P. arguta*. The chloroplast genome of *P. arguta* was sequenced, assembled, and annotated in a previous study [35]. However, the identification of the chloroplast genome in *P. arguta* was incomplete. Therefore, we conducted a comprehensive identification of the chloroplast genome of *P. arguta* and obtained a more complete sequence. In this study, the chloroplast genome of *P. arguta* is primarily divided into the LSC, SSC, and two IR regions, exhibiting a typical quadripartite structure consistent with other reported members of the Hydrangeaceae family [35]. This indicates a high degree of stability in the chloroplast genome structure of the genus *Platycrater*. The total length of the *P. arguta* chloroplast genome is 157,812 bp, with the LSC region measuring 86,825 bp, the SSC region 18,735 bp, and the IR region 26,126 bp. Furthermore, a total of 88 messenger RNA (mRNA) genes, 37 transfer RNA (tRNA) genes, and 8 ribosomal RNA (rRNA) genes have been annotated in the *P. arguta* chloroplast genome, totaling 133 genes, which aligns with the fundamental patterns observed in angiosperm chloroplast genomes.

SSR loci are widely distributed in the genomes of eukaryotes, characterized by significant polymorphism, substantial quantity, and stable inheritance [36,37]. The types, numbers, and distributions of SSRs vary among different plant species, which makes them widely used in molecular marker studies related to species identification and genetic diversity analysis [38,39]. In this study, a total of 199 SSR loci were detected in the chloroplast genome of *P. arguta*, with single nucleotide repeats being the most abundant (119, accounting for 59.8% of the total), followed by trinucleotide repeats (73, 36.68%). These two types of repeats predominantly exist in the forms of A/T and CTT/TTC. The remaining SSRs also mainly consist of combinations of A and T bases, which is consistent with previous observations [36], supporting the notion that SSRs in chloroplast genomes exhibit a significant AT bias, likely related to the easier denaturation of AT compared to GC.

Codon usage bias analysis showed that genes of *P. arguta* tend to have codons with A/T in the third position. This pattern likely reflects the combined effects of evolutionary forces: the strong A/T bias at synonymous sites (28.6% GC3 content) may primarily stem from mutational bias, chloroplast genomes are known to have high A/T content due to inherent mutational tendencies, which is further reinforced by potential selective pressure for translational efficiency (e.g., matching tRNA pools enriched for A/U anticodons, optimizing protein synthesis rate) [40,41]. The pattern of codon usage bias in the plant chloroplast genome is linked to gene structure and expression, which obviously affect gene function [40]. In previous studies, analysis of codon usage bias was conducted to better understand gene characteristics [42]; thus, we speculate this A/T-biased codon usage may modulate *P. arguta* chloroplast gene function by influencing translation efficiency. Pi can reveal the variation in nucleic acid sequences of different species, and regions with a high degree of variation can provide potential molecular markers for population genetics. This study indicates that the highly variable genes in the chloroplast genome of *P. arguta* are primarily located in the SSC and LSC regions. Genes such as *rps15*, *ycf1*, *petN*, *matK*, and *ndhF* have been identified as hypervariable regions across different plants [10].

The contraction and expansion of the IR/SC boundaries play a crucial role in the evolution of chloroplast genomes and are considered the main driving force behind structural variations in chloroplast genomes [43,44,45]. Recent studies (2019–2024) on angiosperm chloroplast genomes further confirm that IR boundary shifts, especially involving genes like ycf1, are common evolutionary events that shape genomic diversity within plant families [44,46]. The analysis of the IR/SC boundaries of the chloroplast genomes from six highly homologous species within the same family selected for this study reveals that the *ycf1* gene is located at the SSC/IRa junction in all seven chloroplast genomes, while the LSC/IRa junction is positioned downstream of trnH-GUG. Notably, *ycf1* holds substantial functional importance that further amplifies interest in its unusual genomic arrangement in *P. arguta*: it is one of the largest open reading frames encoded by the chloroplast genome in higher plants, and together with *ycf2*, experimental studies have demonstrated that both genes are essential for plant viability [47]. This indispensability underscores *ycf1*’s critical role in plastid biology. Evolutionarily, *ycf1* is also significant: its N-terminal sequences are remarkably conserved across more than 700 million years of streptophyte evolution, and even its C-terminal region contains a motif conserved for over 500 million years of streptophyte evolution [48]. The unusual configuration of *P. arguta*’s IR regions, where *ycf1* is largely excluded from the IRs, thus represents a notable divergence from the canonical plastid genome structure, with its functional essentiality making this structural deviation even more worthy of in-depth evolutionary investigation. The JSB and JSA boundaries are relatively conserved, whereas the JLB and JLA boundaries exhibit significant variation, which is consistent with reports on the majority of Hydrangeaceae plants [35].

This study demonstrates that, while plant chloroplast genomes are generally conserved in their four-part structure, *P. arguta* exhibits a distinct bias in configuration. Notably, the functionally important *ycf1* gene is largely excluded from the IR region, indicating that this structural change is not trivial; it suggests that *P. arguta* may have undergone evolutionary or functional adaptations to preserve *ycf1* function despite this genomic rearrangement. This phenomenon is not isolated, as similar occurrences have been observed in other species [47,48], providing a new perspective for understanding the constraints and flexibility in the evolution of plastid genomes.

Previous studies suggest that species with closer phylogenetic relationships exhibit more similar boundary features [49,50,51]. Phylogenetic trees constructed for 22 species (under GTRGAMMA model with 1000 bootstrap replicates) indicate that *P. arguta* forms a well-supported monophyletic clade with *H. davidii* (bootstrap support = 100%), and the pairwise sequence identity between their complete chloroplast genomes is 98.7%, the highest among all compared taxa. *P. arguta* and *H. davidii* together cluster with 17 other Saxifragaceae species, with most internal nodes receiving bootstrap support > 90%. Our comparative analysis aligns with these previous findings, demonstrating that the complete chloroplast genome of *P. arguta* is conserved [52].

## 5. Conclusions

In this study, we analyzed the chloroplast genome of *P. arguta* and found that it exhibits a typical quadripartite structure, with a genome size of 157,812 bp and a total of 133 annotated genes. SSRs detection indicated that the chloroplast genome includes 199 simple repeat loci, with the highest frequency of mononucleotide repeats composed of A and T, accounting for 59.8%. Comparative analysis of the chloroplast genome structure revealed that the IR region boundaries are relatively conserved. Phylogenetic analysis showed that *P. arguta* shares the highest homology with *H. davidii*. The findings provide a scientific basis for future research on the genetics, biology, and conservation factors of *P. arguta* and other endangered plants.

## Figures and Tables

**Table 1:** Gene contents of the *P. arguta* chloroplast genome based on genome annotation.

Category	Gene Group	Gene Name
Photosynthesis	Subunits of photosystem I	*psaA*, *psaB*, *psaC*, *psaI*, *psaJ*
	Subunits of photosystem II	*psbA*, *psbB*, *psbC*, *psbD*, *psbE*, *psbF*, *psbH*, *psbI*, *psbJ*, *psbK*, *psbL*, *psbM*, *psbN*, *psbT*, *psbZ*
	Subunits of NADH dehydrogenase	*ndhA*, *ndhB (2)*, *ndhC*, *ndhD*, *ndhE*, *ndhF*, *ndhG*, *ndhH*, *ndhI*, *ndhJ*, *ndhK*
	Subunits of cytochrome b/f complex	*petA*, *petB*, *petD*, *petG*, *petL*, *petN*
	Subunits of ATP synthase	*atpA*, *atpB*, *atpE*, *atpF*, *atpH*, *atpI*
	Large subunit of rubisco	*rbcL*
Self-replication	Proteins of large ribosomal subunit	*rpl14*, *rpl16*, *rpl2 (2)*, *rpl20*, *rpl22,* *rpl23(2)*, *rpl32*, *rpl33*, *rpl36*
	Proteins of small ribosomal subunit	*rps11*, *rps12 (2)*, *rps14*, *rps15*, *rps16*, *rps18*, *rps19*, *rps2*, *rps3*, *rps4*, *rps7(2)*, *rps8*
	Subunits of RNA polymerase	*rpoA*, *rpoB*, *rpoC1*, *rpoC2*
	Ribosomal RNAs	*rrn16(2)*, *rrn23(2)*, *rrn4.5(2)*, *rrn5(2)*
	Transfer RN	*trnA-UGC (2)*, *trnC-GCA*, *trnD-GUC*, *trnE-UUC*, *trnF-GAA*, *trnG-GCC*, *trnG-UCC*, *trnH-GUG*, *trnI-CAU(2)*, *trnI-GAU (2)*, *trnK-UUU*, *trnL-CAA(2)*, *trnL-UAA*, *trnL-UAG*, *trnM-CAU*, *trnN-GUU(2)*, *trnP-UGG*, *trnQ-UUG*, *trnR-ACG(2)*, *trnR-UCU*, *trnS-GCU*, *trnS-GGA*, *trnS-UGA*, *trnT-GGU,* *trnT-UGU*, *trnV-GAC(2)*, *trnV-UAC*, *trnW-CCA*, *trnY-GUA*, *trnfM-CAU*
Other genes	Maturase	*matK*
	Protease	*clpP*
	Envelope membrane protein	*cemA*
	Acetyl-CoA carboxylase	*accD*
	c-type cytochrome synthesis gene	*ccsA*
	Translation initiation factor	*infA*
Genes of unknown function	Conserved hypothetical chloroplast ORF	*ycf1(2)*, *ycf15(2)*, *ycf2(2)*, *ycf3*, *ycf4*

## Data Availability

The genome sequence data that support the findings of this study are openly available in GenBank of NCBI at https://www.ncbi.nlm.nih.gov/ (accessed on 1 February 2024) under the accession number OL405445. The associated “BioProject,” “SRA,” and “Bio-Sample” numbers are PRJNA774471, SRR16572822, and SUB10569978, respectively.

## Supplementary Materials

The following supporting information can be downloaded at: https://www.mdpi.com/article/10.3390/biology14121726/s1, Table S1: The repeats in the *P. arguta* chloroplast genome.

## Abbreviations

Bp	Base pair
LSC	Large single copy
SSC	Small single copy
IR	Inverted repeat
IUCN	International Union for Conservation of Nature
A	Adenine
T	Thymine
G	Guanine
C	Cytosine
Leu	Leucine
Cys	Cysteine
RSCU	Relative synonymous codon usage
SSR	Single sequence repeat
PEP	Plastid-encoded plastid RNA polymerase
NEP	Nucleus-encoded plastid RNA polymerase
DNA	Deoxyribonucleic acid
