# Supplementary material for: Complete Chloroplast Genome Characterization, and Phylogenetic Analyses of the Rare and Endangered Plant Platycrater arguta
Source: Biology (Basel). 2025 Dec 1;14(12):1726. doi: 10.3390/biology14121726 (PMC12730320; doi:10.3390/biology14121726)
Supplement: Supplementary file 1 [file biology-14-01726-s001.zip › biology-3915104-supplementary.pdf]

Table S1. The repeats in the *P. arguta* chloroplast genome

Id	LengthI	Start I	Type	LengthII	Start II
1	26126	86826	P	26126	131687
2	58	94098	F	58	94116
3	58	94098	P	58	150465
4	58	94116	P	58	150483
5	58	150465	F	58	150483
6	56	79344	P	56	79344
7	53	118751	P	53	118751
8	51	94108	F	51	94126
9	51	94108	P	51	150462
10	51	94126	P	51	150480
11	48	31003	P	48	31003
12	45	150478	F	45	150496
13	44	76902	P	44	76902

14	42	101 191	F	42	123 517
15	42	123 517	P	42	143 406
16	42	451 32	F	42	123 516
17	42	940 96	F	42	941 32
18	42	940 96	P	42	150 465
19	42	941 32	P	42	150 501
20	42	150 465	F	42	150 501
21	40	941 16	F	40	941 34
22	40	941 16	P	40	150 465
23	40	941 34	P	40	150 483
24	39	451 35	F	39	101 193
25	39	451 35	P	39	143 407
26	33	941 08	F	33	941 44
27	33	113 375	F	33	113 402
28	31	900	F	31	369

		2			75
29	31	915<br>8	R	31	916<br>0
30	30	900<br>3	P	30	468<br>29
31	30	141<br>19	P	30	141<br>19
32	30	369<br>76	P	30	468<br>29
33	30	401<br>42	F	30	423<br>65
34	30	451<br>47	F	30	101<br>205
35	30	451<br>47	P	30	143<br>404
36	30	819<br>67	F	30	819<br>91
37	30	916<br>54	F	30	916<br>96
38	30	916<br>54	P	30	152<br>913
39	30	916<br>96	P	30	152<br>955
40	30	150<br>478	F	30	150<br>514
41	30	152<br>913	F	30	152<br>955
